# Simultaneous fMRI-EEG-Based Characterisation of NREM Parasomnia Disease: Methods and Limitations

**DOI:** 10.3390/diagnostics10121087

**Published:** 2020-12-14

**Authors:** Marek Piorecky, Vlastimil Koudelka, Eva Miletinova, Jitka Buskova, Jan Strobl, Jiri Horacek, Martin Brunovsky, Stanislav Jiricek, Jaroslav Hlinka, David Tomecek, Vaclava Piorecka

**Affiliations:** 1 National Institute of Mental Health, 25067 Klecany, Czech Republic; vlastimil.koudelka@nudz.cz (V.K.); eva.miletinova@nudz.cz (E.M.); jitka.buskova@nudz.cz (J.B.); jiri.horacek@nudz.cz (J.H.); stanislav.jiricek@nudz.cz (S.J.); jaroslav.hlinka@nudz.cz (J.H.); david.tomecek@nudz.cz (D.T.); vaclava.piorecka@nudz.cz (V.P.); 2 Department of Biomedical Technology, Faculty of Biomedical Engineering, CTU in Prague, 27201 Kladno, Czech Republic; jan.strobl@fbmi.cvut.cz; 3 Third Faculty of Medicine, Charles University, 10000 Prague, Czech Republic; 4 Institute of Computer Science of the Czech Academy of Sciences, 18207 Prague, Czech Republic; 5 Department of Cybernetics, Faculty of Electrical Engineering, Czech Technical University in Prague, 16627 Prague, Czech Republic

**Keywords:** fMRI, EEG, simultaneous measurement, disorders of arousal, NREM parasomnia

## Abstract

Functional magnetic resonance imaging (fMRI) techniques and electroencephalography (EEG) were used to investigate sleep with a focus on impaired arousal mechanisms in disorders of arousal (DOAs). With a prevalence of 2–4% in adults, DOAs are significant disorders that are currently gaining attention among physicians. The paper describes a simultaneous EEG and fMRI experiment conducted in adult individuals with DOAs (n=10). Both EEG and fMRI data were validated by reproducing well established EEG and fMRI associations. A method for identification of both brain functional areas and EEG rhythms associated with DOAs in shallow sleep was designed. Significant differences between patients and controls were found in delta, theta, and alpha bands during awakening epochs. General linear models of the blood-oxygen-level-dependent signal have shown the secondary visual cortex and dorsal posterior cingulate cortex to be associated with alpha spectral power fluctuations, and the precuneus with delta spectral power fluctuations, specifically in patients and not in controls. Future EEG–fMRI sleep studies should also consider subject comfort as an important aspect in the experimental design.

## 1. Introduction

Sleep and its disorders are at the forefront of many researchers’ and physicians’ interests. Generally, sleep disturbances have been reported by 30% of the population. Sleep disorders may cause or exacerbate preexisting medical and psychiatric conditions and are associated with high rates of depression, anxiety, and impaired daytime functioning [1]. Sleep is the relaxation phase. Muscles relax, respiratory and cardiac activity change, brain activity changes, and so does the state of consciousness. Sleep contains cycles alternating in several phases with different characteristics. The main two types of sleep are called the rapid eye movement (REM) and non-rapid eye movement (NREM) phases. By default, we can further divide the NREM phase into three parts according to the depth of sleep, with the third being the deepest [2]. Sleep, like all physiological processes, can be disturbed. Some of the most prevalent sleep disorders are disorders of arousal (DOAs; sleepwalking, confusional arousal, and night terrors). DOAs are the NREM parasomnias characterised by abnormal behavioural (motor or verbal) events during NREM sleep. People with DOAs can have reduced quality of sleep that affects their health [3,4].

Sleepiness in adults can have a major impact on a daily routine. Studies show that the primary cause of DOA episodes is the instability of the NREM phase of sleep [5,6]. During a parasomnic attack, the activity of the slow waves in the EEG decreases during the first sleep cycle, and those waves are more evenly distributed across the time in the signal [7,8].

Advances in understanding physiological and pathological processes in the brain are directly dependent on the methodological approach used. Electroencephalography (EEG) and functional magnetic resonance imaging (fMRI) are very effective tools for characterising human brain function. DOAs have been studied by EEG, where they are associated with activation in the frontal lobe and with synchronised slow wave activity before the onset of the parasomnic episode [4,5,9,10]. Manifestations of DOAs were also examined by fMRI [11] and the positron emission tomography [12]. Simultaneous sleep recordings using EEG and fMRI have been described in studies [13,14,15,16,17], but without specifically focusing on parasomnia. Sleep is usually studied by well established EEG components which are, however, originated in deep brain sources. The fMRI modality is suitable for precise localisation of activity in the whole brain, so the EEG and fMRI data integration is a promising tool for studying the spatio-temporal dynamics of brain activity. To the best of our knowledge, the most promising potential of combined EEG and fMRI recording has so far not been utilised in parasomnia research. Our research exploited recent EEG–fMRI data analysis techniques and resulted in sleep research with the aim of better characterising DOAs.

Simultaneous EEG and fMRI recording, however, brings a number of limitations. At first, the recording in an MRI is inappropriate for subjects with claustrophobia. Larger discomfort in simultaneous recording (EEG electrodes located on head, noise, and the constant position of the body in an MRI scanner during recordings) requires special design of sleep experiments. Furthermore, sleep deprivation has to be applied to allow the subject to fall asleep, despite the subject’s discomfort. However, long sleep deprivation can lead to non-physiological sleep during the recording session, which can lead to invalid results. The time of sleep deprivation before the start of a recording varies across studies. For example, in the study [18], the sleep deprivation was 17 h; in studies [13,19], it was 36 h.

Recording time limitations due to the EEG and fRMI equipment is another significant issue of sleep experiments utilising simultaneous and continuous recording of EEG and fMRI. There are two main parameters that influence the maximal continuous recording time. The first one is the maximum number of MRI scans, defined for a specific MRI sequence, which is by default set by the Siemens Magneton Prisma system. The next limiting parameter is the EEG amplifier’s battery capacity. Generally, these two parameters do not allow continuous overnight recording. Hong et al. recorded simultaneous EEG and fMRI throughout the entire night, but the recordings were discontinuous [20]. Moehlman et al. continuously recorded EEG and fMRI sleep data throughout the entire night [16]; however, how the continuous recording was achieved was not specified in detail.

Beside the above-mentioned technical limitations, a variety of technical and physiological artefacts are propagated in both modalities EEG and fMRI throughout the simultaneous recording approach. The artefact occurrence can be partially reduced by an appropriate setup of the EEG and fMRI. Chowdhury et al. examined the EEG lead’s influence on the genesis EEG artefacts [21]. It has been proved that the gradient artefact (GA) generated by the ribbon cable is greater than the GA generated by a twisted cable. Chowdhury et al. mentioned that there are higher GA changes using a ribbon cable if the lead position changes. The results of this study showed that twisted cables placed in a constant magnetic field direction are preferable [21].

The MRI environment itself causes a number of substantial artefacts in the EEG data. If those artefacts are not sufficiently suppressed, the analysis can lead to false positive statistical significance. Among those, the two most prominent are GA and pulse (PA) artefacts. Another essential part of artefact suppression is the use of appropriate preprocessing methods. The suppression of those artefacts is typically based on the fact that they are relatively stable over time. Thus, the average artefact subtraction method (AAS) combined with principal component analysis (PCA) or independent component analysis (ICA) decomposition, called the optimal basis set (OBS), is typically utilised for both GA [22,23,24] and PA [25] artefact suppression. Those methods are conveniently implemented in EEGLab plug-in fMRIB [26]. The PA artefact is the most challenging phenomenon contaminating the lower bands of an EEG, and these bands are of interest in sleep research [27]. Here, we utilised the aOBS algorithm [27] exhibiting the most effective artefact suppression compared to the previously used approaches in sleep. Furthermore, we validated the data quality using our recent algorithm based on an application of a machine learning clustering algorithm called t-distributed stochastic neighbour embedding (t-SNE) [28].

For the purpose of EEG and fMRI data integration, one has to ensure that the EEG equipment does not influence the blood-oxygen-level-dependent (BOLD) signal in terms of producing an EEG-specific BOLD artefact. Previous EEG–fMRI studies in sleep have not addressed this phenomenon in sufficient detail. Here, we validated the BOLD signal by deriving well established resting state networks from the awake epochs during the recording session.

The aim of the study was to compare the sleep of NREM parasomnic individuals and a control group using simultaneously recorded hdEEG and fMRI. We present a methodology based on the available literature on EEG–fMRI in the sleep of healthy human subjects, and we propose mathematical tools to evaluate the quality of EEG and fMRI data in sleep. The first goal of this study is to prove that it is possible to simultaneously record all sleep stages in NREM parasomnia patients. The second goal was to validate the preprocessed and integrated simultaneous EEG–fMRI data. Most importantly, the paper answers the question of whether there are significant differences between parasomnia patients and a control group according to the introduced hdEEG–fMRI integration approach.

## 2. Materials and Methods

This section describes all the methods utilised in this study and is divided into 9 subsections. The first Section 2.1 describes the complete experimental design. Section 2.2 summarises all the equipment utilised in this study together with MR scanner acquisition parameters. Section 2.3 and Section 2.4 include EEG and fMRI preprocessing methodology and their evaluation. Section 2.5 and Section 2.6 describe the integration approach itself on the electrode level and fMRI volume level, respectively. Finally, Section 2.7, Section 2.8 and Section 2.9 involve statistical testing. All scripts are freely available online (https://github.com/MPio23/SimultEEG_fMRI).

### 2.1. Experiment Design

Patients with NREM parasomnia and healthy subjects (control group) were compared during the experiment. All the patients met the ICSD-3 criteria for SW (sleepwalking) and for ST (sleep terror). Every study subject underwent a clinical interview performed by a neurologist specialised in sleep medicine and overnight video-polysomnography in the National Institute of Mental Health (NIMH). The absence of exclusion criteria was verified before the start of the experiment for each subject; see Table 1.

**Figure 1:**
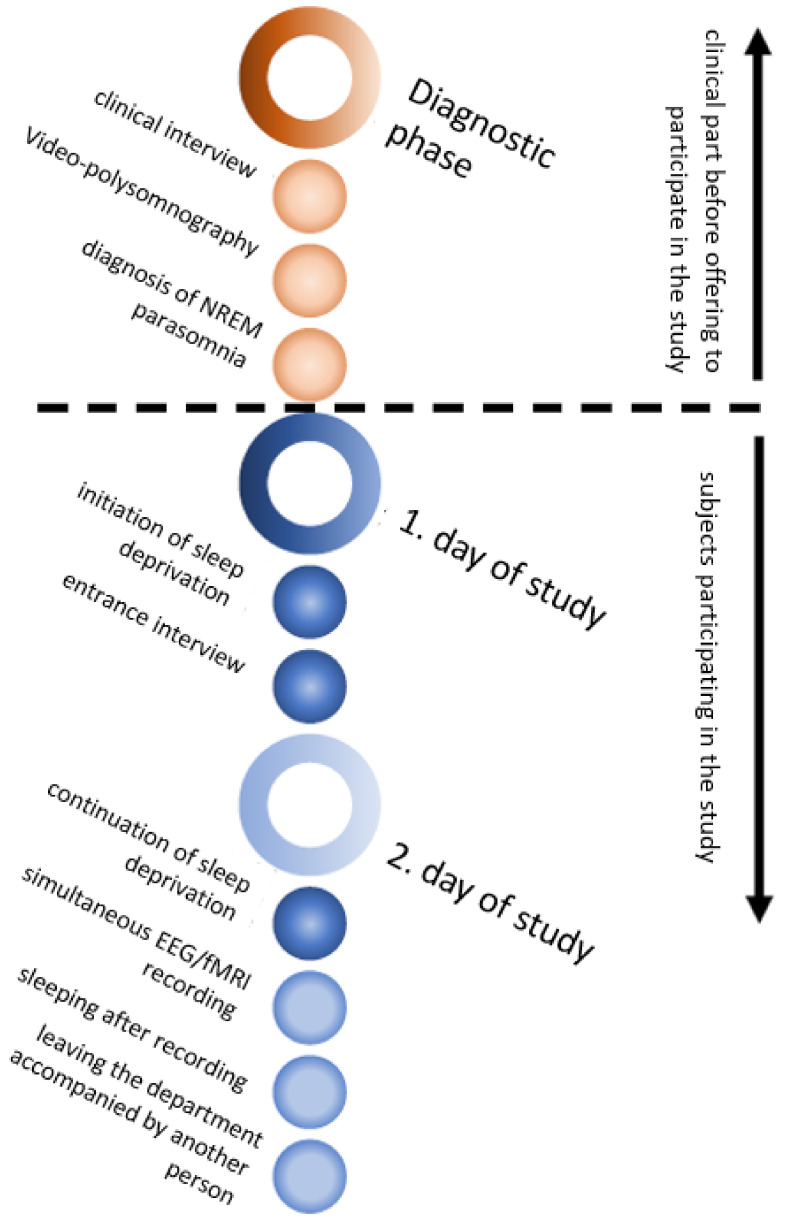
The diagram (time goes from top to bottom) shows the time schedule of the study, including the volunteer selection phase.

In our study, subjects were asked in advance whether they have had any kind of claustrophobic experience in the past. Subjects underwent sleep deprivation for 28 h ± 1 h. The subject got up in the morning according to his habits (no later than 8:00), a normal working day took place, and at 17:00 he arrived at the sleep laboratory in NIMH. Subjects were under control all night, and a supine position was not allowed. An examination by a physician was performed in the morning, and the measurement itself was performed around lunch time. The entire process is summarised in a timeline of the entire study in Figure 1. Small pillows were used to fix the subject’s head and increase the comfort of the subject during a recording session. An EEG cable harness below the subject’s body was placed in accordance with subject comfort. We emphasised the subject’s comfort over minimising the GA artefact, which was further removed by an appropriate preprocessing technique. A solution to this trade-off was designed for future studies; see Appendix A. We utilised an 83-min-long fMRI series that allowed us to reliably involve the first sleep circle. Despite the control of subject comfort, most of the recordings did not contain enough NREM sleeping phases; see Section 4.

We recorded 10 subjects in the control group and the group with patients with NREM parasomnia. This dataset is very unique in the specific experimental design and the types of disease studied.

Simultaneous recordings from 8 control subjects and 9 patients with NREM parasomnia remained for subsequent data processing after the exclusion of wrong datasets (one control subject was proven to present symptoms of patients with NREM parasomnia retrospectively; two recordings had to be excluded due to technical issues during recording). Sleep phases and transitions to sleep and awakening were scored by two experts in accepted datasets. A third expert scored unclear parts of the data when two experts did not find a match.

The study protocol and patient informed consent were approved by the National Institute of Mental Health’s ethical committee on 9th of August, approval code: 185/17. The procedures followed were in compliance with the ethical standards of the responsible committee on human experimentation (institutional and national) and with the World Medical Association’s Declaration of Helsinki on Ethical Principles for Medical Research Involving Human Subjects.

### 2.2. Technical Equipment

The MR compatible Geodesic EEG System (GES) 400 from Electrical Geodesics, Inc., Eugene, OR, USA (EGI) was used to measure EEG. The system included a Net Amps 400 amplifier, which was controlled by an iMac computer with Net Station software; synchronisation was controlled by the GES Clock Sync I/O device. The system versions used are described in Table 2. The EEG measurement cap was used by the same company MR-compatible HCGSN (HydroCel Geodesic Sensor Net) 256 channels (see the last row of Table 2). Due to the length of the measurement, a hydrogel-based material was used. EEG data were recorded with a sampling frequency of 1 kHz.

An MR device with a static magnetic field size of 3T from Siemens, model Siemens Magneton Prisma, was used for the measurement. The sequence parameters used for fMRI sleep recording are described in Table 3.

### 2.3. EEG Preprocessing

First of all, the EEGlab plug-in FMRIB was utilised. The FASTR algorithm, which is based on the average artefact subtraction (AAS) method, was applied to EEG data to suppress the gradient artefact [23,26]. To remove the ballistocardiographic artefact from the EEG data, the adaptive optimal basis set (aOBS) algorithm was utilised [27]. Subsequently, the eye artefact was removed from the data based on a method in the Fieldtrip toolbox [29,30]. Finally, a notch filter was applied, and the data were then band-pass-filtered between 0.5 and 30 Hz and referenced to the average signal obtained from two electrodes corresponding to mastoids, which is the most typical electrode reference in studies dealing with sleep data. For validation of preprocessed EEG data, our unsupervised clustering algorithm based on tSNE was applied to the data, and resulting clusters were visually checked and compared with resting-state datasets utilised in our previous study [28].

### 2.4. fMRI Preprocessing

The BOLD data were preprocessed by a pipeline consisting of the following steps: a bias field correction, motion correction, slice-timing correction, normalisation, and spatial smoothing. All the steps were performed in the SPM toolbox [31]. The bias field correction is not a standard step in the BOLD data preprocessing. This step is included with the pipeline to utilise a 64-channel coil that is sensitive to intensity inhomogeneity in recorded signals. Typically, this step is performed directly within the MR scanner post-processing utilities. If this is not done, it can be performed as a first step in the preprocessing itself, e.g., using the segmentation tool in SPM and estimating a bias field. The bias field is then used to correct the data, as in our case.

For the bias field correction, the regularisation parameter was set to 0.001, and the FWHM parameter was set to 60. These are the segmentation parameters, which represent an auxiliary step in our bias field correction method. Subsequently, all volumes were realigned by a rigid body transformation to the average volume, which was chosen as a reference volume.. Finally, volumes were normalised to the MNI space and smoothed by a Gaussian of 8 × 8 × 8 voxel size. Translation and rotation parameters were utilised as additional regressors to a GLM. As a validation part of the fMRI preprocessing, the part of the BOLD data labelled by a specialist as a resting-state was merged in time and subjected to a spatial ICA to visually check whether a set of resting-state networks can be obtained.

### 2.5. Spectral Analysis

Spectrum estimation was performed using multitaper frequency transformation [32]. The Slepian window (discrete prolate spheroidal sequences, DPSS), which maximised the energy concentration in the main lobe and averaged the noise in the spectrum of [32,33], was used. The frequency bands were defined as follows: delta (0.5–4.5 Hz), theta (4.5–8.5 Hz), alpha (8.5–13.0 Hz), and beta (13.0–30.0 Hz). The obtained absolute power spectrum was normalised. The individual spectral bands were divided by the total power (sum over all bands). The resulting relative spectra of individuals then ranged from 0 to 1, allowing for interindividual comparisons. Normalisation also suppresses, for example, differences in the conductivity of the skull [34]. Permutation tests (Monte Carlo estimates of the probability of significance and critical values) of the FieldTrip toolbox for MATLAB [35] were used for statistical evaluation of normalised power spectra. The tested dataset contained time series from 256 electrodes from each subject. The multiple comparisons were corrected by the cluster method [35].

### 2.6. EEG and Sleep Covariates

As it was not possible to detect standard sleep phases in the EEG recordings, physicians identified sections of the EEG that corresponded to transitions to sleep and awakening. The presence of a given phase (A/T) was indicated in the occurrence vectors by ones and zeros. These vectors (A/T) were convolved with a hemodynamic response function. The vectors of spectral power fluctuations over time (for all bands) were convolved with the hemodynamic response function. With regard to the size of the dimension—for one subject, 256 vectors (corresponds to the number of channels)—an appropriate method for reducing the dimension of selected electrodes was chosen. In previous papers, several linear [36,37] and non-linear [38,39,40] dimension reduction methods were utilised directly to EEG data. Nevertheless, it is still unknown what combination of EEG features and the dimension reduction technique reliably describes the BOLD signal and if the association is linear or not. Since it was shown in [4] that parasomnia is connected with EEG high-beta power activation in cingulate motor areas, the band-limited power (BLP) fluctuations of an EEG can be considered as a robust feature for hdEEG–fMRI data integration. In this paper, BLP-based integration was validated by deriving known correspondence between fluctuations of BLP in an EEG alpha band and the BOLD signal in occipital regions. Electrodes were selected based on previous statistics for each frequency band separately, with an emphasis on differentiating patients and controls. Electrodes belonging to the most significant cluster are marked. At the same time, the part in which subjects were awake just after being placed in the MR scanner was always recorded. This part was used to validate the BOLD preprocessing pipeline and to orthogonalise BOLD components of awake epochs in GLMs.

### 2.7. First Level Statistic

The GLM is a compact way to write multiple regression models. It can be considered as an extension of linear regression. Linear regression is the method used to calculate the trend that best approximates the set of experimental data. In linear regression, the measured BOLD signal intensity data are plotted on the y axis, and the stimulus for which the response is sought on the x axis. For the voxel and each data point with coordinates $\left[x_{i},y_{i}\right]$, the following relation can be written:

(1) $$y_{i}=x_{i}\cdot \beta +\epsilon_{i},$$

where β is the calculated slope of the line, and $\epsilon_{i}$ is the calculated error, or residual. The residue corresponds to the distance between the line and the corresponding data point. The values of $x_{i}$ are defined by the experiment itself, the values of $y_{i}$ are measured, while the parameters β and $\epsilon_{i}$ are calculated. Matrix *X* containing the experiment information is usually larger (it is not just one vector). Each *X* column reflects a specific factor that is assumed to affect the outcome of the experiment. In this study, the last seven columns are made up of regressors describing the interference. There are translations and rotational movements in three directions and a regressor representing an increased hemodynamic response to placement in the MR scanner.

The resulting values of the β parameters can then enter statistical tests, where it is possible to test hypotheses about the covariates themselves or their combinations. In the first level of statistics, the activations for each subject are compared separately. The extracted models for each individual then enter the second level of statistical testing for cross-group comparisons.

The final output is usually a statistical parametric map, which often overlaps with an anatomical image or a template made up of average images when the results are displayed.

### 2.8. Second-Level Statistic

Statistical parametric mapping (SPM) is the most common approach to characterise functional changes and changes related to brain pathology. SPM displays the result of statistical analyses for the GLM group/groups. It corresponds to beta coefficient contrasting images summarising the effects for each subject (see Equation (1)). This approach is implemented in the tool of the same name in the MATLAB environment. A version of this software SPM12 [31] was used in this study. This is statistical testing for each voxel followed by multiple comparison corrections. Statistical parametric maps are images with voxel values, which are distributed according to the null hypothesis according to the known probability density function, according to the T distribution. The results are colour-coded on maps with anatomical images. The *p* value was not corrected for multiple comparisons due to the high number of voxels (corrected *p* value threshold in the order of 10${}^{-7}$). Highlighted areas represent areas of the brain or voxels that are thought to have statistically significant activations or correlations with given covariates at a significant level of 0.01 or 0.05. A significant level represents the maximum error we can have in statistical testing. A significance level of 0.05 was used in case of low sensitive results. The reason is, that the significance level of 0.01 can suppress substantial results with low sensitivity. Both settings of significant level are used in the studies with simultaneous measuring EEG/fMRI. Post-statistical correction was performed by setting the threshold for the minimum number of voxels in the cluster, thus eliminating spatially insignificant activations.

In order to answer the hypotheses concerning joint activation, an “intersection” approach was proposed. This approach involves the extraction of activation based on the second-level statistics of both groups (patients and controls) and the subsequent comparison of areas where there is an intersection in the statistical parametric map for both groups.

### 2.9. Hypotheses and Statistical Evaluation

The first main hypothesis was focused on comparing the patients with NREM parasomnia with control subjects in deep sleep. This hypothesis had to be changed due to small occurrences of deep sleep phases in the records scored by experts (see Section 3.1). The results of scoring showed that transitions to sleep and awakening occurred more often in the recorded datasets. The awakening phase was joined with a parasomnic effect of sleep disturbation [7]. The new hypotheses were focused on comparing patients with NREM parasomnia with control subjects under T and A conditions, for this reason. The study tested several modified hypotheses regarding the relationship between EEG, sleep covariates (A and T), and spontaneous BOLD fluctuations.

Complete list of the tested hypothesis:

1. Awakening (A) covariates vs. BOLD
  (a)
    **Hypothesis** **1** **(H1).** The awakening regressor in the GLM control group does not correlate with BOLD fluctuations.
  (b)
    **Hypothesis** **2** **(H2).** The awakening regressor in the GLM patient group does not correlate with BOLD fluctuations.
  (c)
    **Hypothesis** **3** **(H3).** The awakening regressor for the patient and control groups in the GLM has no common region activation (intersection methodology—not a significance level).
2. Transition to a sleep (T) covariates vs. BOLD
  (a)
    **Hypothesis** **4** **(H4).** The transition to a sleep regressor in the GLM control group does not correlate with BOLD fluctuations.
  (b)
    **Hypothesis** **5** **(H5).** The transition to a sleep regressor in the GLM patient group does not correlate with BOLD fluctuations.
  (c)
    **Hypothesis** **6** **(H6).** The transition to a sleep regressor for the patient and control groups in the GLM has no common region activation (intersection methodology—not a significance level).
3. BLP covariates vs. BOLD in alpha band
  (a)
    **Hypothesis** **7** **(H7).** The alpha BLP regressor in the GLM control group does not correlate with BOLD fluctuations.
  (b)
    **Hypothesis** **8** **(H8).** The alpha BLP regressor in the GLM patient group does not correlate with BOLD fluctuations.
  (c)
    **Hypothesis** **9** **(H9).** The alpha BLP regressor for the patient and control groups in the GLM has no common region activation (intersection methodology—not a significance level).
4. BLP covariates vs. BOLD in theta band
  (a)
    **Hypothesis** **10** **(H10).** The theta BLP regressor in the GLM control group does not correlate with BOLD fluctuations.
  (b)
    **Hypothesis** **11** **(H11).** The theta BLP regressor in the GLM patient group does not correlate with BOLD fluctuations.
  (c)
    **Hypothesis** **12** **(H12).** The theta BLP regressor for the patient and control groups in the GLM has no common region activation (intersection methodology—not a significance level).
5. BLP covariates vs. BOLD in delta band
  (a)
    **Hypothesis** **13** **(H13).** The delta BLP regressor in the GLM control group does not correlate with BOLD fluctuations.
  (b)
    **Hypothesis** **14** **(H14).** The delta BLP regressor in the GLM patient group does not correlate with BOLD fluctuations.
  (c)
    **Hypothesis** **15** **(H15).** The delta BLP regressor for the patient and control groups in the GLM has no common region activation (intersection methodology—not a significance level).
6. BLP EEG in case of T conditions
  (a)
    **Hypothesis** **16** **(H16).** Alpha power fluctuations under T conditions have the same character in the patient and control groups.
  (b)
    **Hypothesis** **17** **(H17).** Beta power fluctuations under T conditions have the same character in the patient and control groups.
  (c)
    **Hypothesis** **18** **(H18).** Delta power fluctuations under T conditions have the same character in the patient and control groups.
  (d)
    **Hypothesis** **19** **(H19).** Theta power fluctuations under T conditions have the same character in the patient and control groups.
7. BLP EEG in case of A conditions
  (a)
    **Hypothesis** **20** **(H20).** Alpha power fluctuations under A conditions have the same character in the patient and control groups.
  (b)
    **Hypothesis** **21** **(H21).** Beta power fluctuations under A conditions have the same character in the patient and control groups.
  (c)
    **Hypothesis** **22** **(H22).** Delta power fluctuations under A conditions have the same character in the patient and control groups.
  (d)
    **Hypothesis** **23** **(H23).** Theta power fluctuations under A conditions have the same character in the patient and control groups.
8. BLP EEG in case of Th-A difference
  (a)
    **Hypothesis** **24** **(H24).** Alpha power fluctuations under difference Th-A conditions have the same character in the patient and control groups.
  (b)
    **Hypothesis** **25** **(H25).** Beta power fluctuations under difference Th-A conditions have the same character in the patient and control groups.
  (c)
    **Hypothesis** **26** **(H26).** Delta power fluctuations under difference Th-A conditions have the same character in the patient and control groups.
  (d)
    **Hypothesis** **27** **(H27).** Theta power fluctuations under difference Th-A conditions have the same character in the patient and control groups.

Each hypothesis on GLM was tested by a single-sample t-test. The results are shown without corrections for multiple comparisons, at a significance level of 0.01, and in the case of lower sensitivity, at a significance level of 0.05. Hypotheses concerning the comparison of spectral power fluctuations in EEG were tested by a permutation test and corrected using the cluster method.

The tool [41] in MATLAB was used to display significantly activated areas. This tool also includes the possibility for export pronounced anatomic areas from the automated anatomical atlas (AAL) and the Brodmann labels corresponding to the statistically significant cluster. The exported table contains, for each significant cluster, its spatial distribution over given anatomical structures and the number of voxels belonging to the cluster that fall into the given anatomical area. At the same time, with this tool, it is possible to display multiple resulting activations over each other for an average brain template.

## 3. Results

The results are divided into five parts in the manuscript. The first subsection answers the question of the implementation of EEG and fMRI sleep recording. The second subsection describes the validation of the quality of the measured data. The last three subsections reflect approaches to finding differences in brain behaviour in NREM parasomnic individuals.

### 3.1. Characterised Limitation of the Sleep Simultaneous Recording

**Figure 2:**
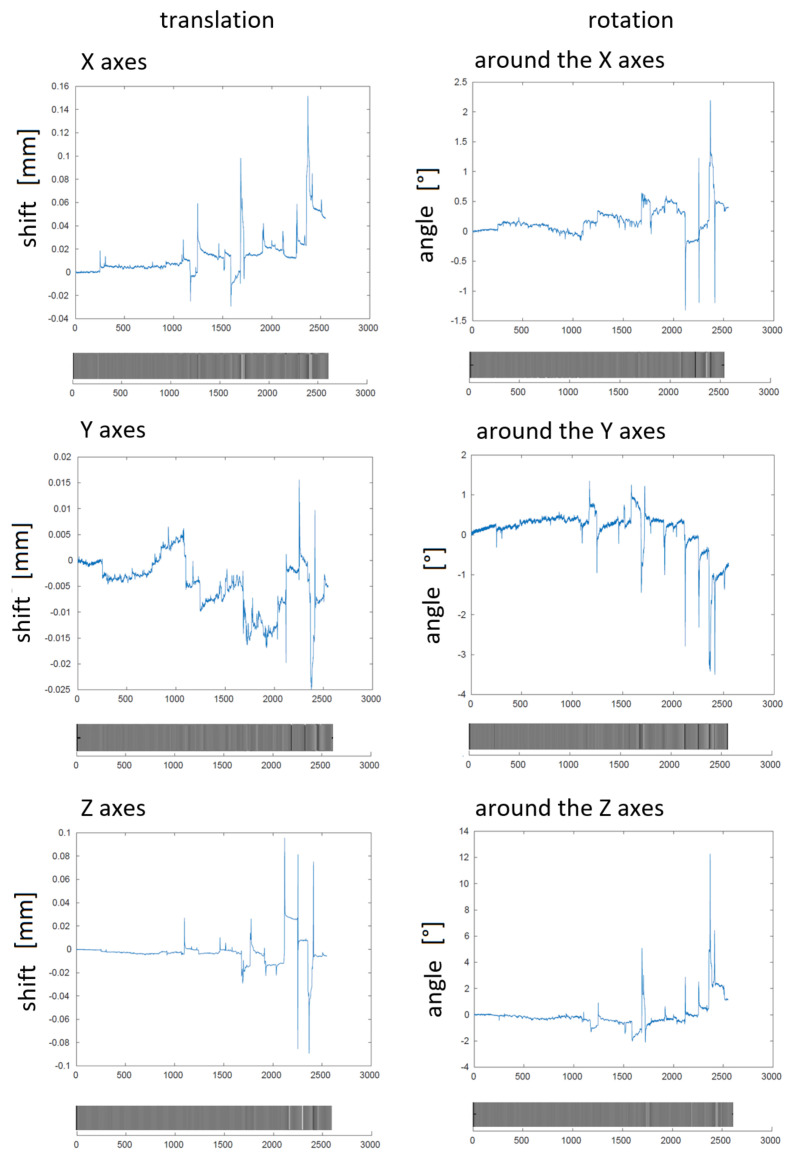
Example of the human head translation (axes x, y, and z) in millimetres and the human head rotation (around axes x, y, and z) in degrees. Grayscale figures show the same information. Moving of the human head is displayed after the first correction by the SPM toolbox and points to an awakening of the subject.

When scoring sleep, it was found that the measured subjects had problems falling into a deep sleep. This may be caused by a combination of several factors. The first one we reflect is a growing feeling of constraint in the space of the MR gantry. Figure 2 shows head translation and rotation over time during simultaneous recording estimated by the method for correction. This correction was performed using the commonly used function spm12_realign from the SPM toolbox [31]. The figure shows that, with a longer recording time, there was a greater movement of the head, associated with increasing discomfort leading to awakening at the end of the recording.

**Figure 3:**
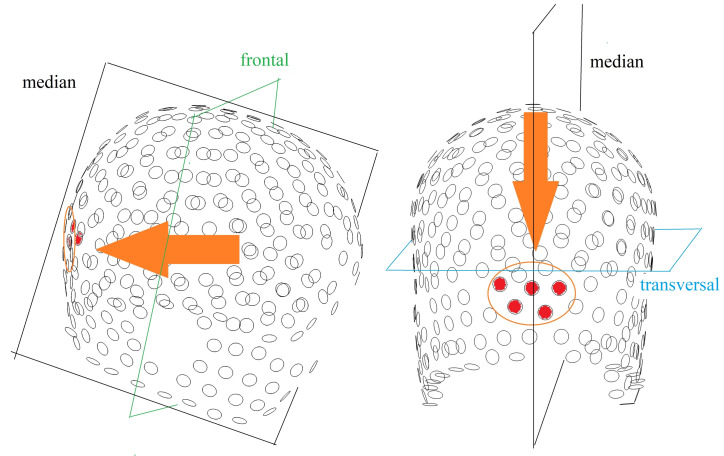
Spatial mesh of the 256 EEG electrodes from the company EGI with marked electrodes (red) which subjects described as uncomfortable. The figure shows the mesh from the right temporal site (**left**) and the occipital site (**right**).

Most of the subjects complained of the increasing pressure of the electrodes in the occipital region (see Figure 3) over time. Lying on the occipital electrodes could cause this most prominent discomfort during simultaneous recording. This discomfort, which can become pain, woke up the subjects during the experiment. Control subjects were asked to locate the dominant area of electrode pressure. Electrodes causing unpleasant pressure are marked in red in Figure 3.

Due to the discomfort issue described above, a sufficient amount of reliable deep sleep data was not available for statistical assessment of the difference between the patients with parasomnia and the control group in deep sleep. The research question was adjusted to find the difference between patients with parasomnia (specifically NREM) and those in the control groups in the transitions to sleep and awakening for this reason (see Section 2.9). Table 4 shows the duration and number of transitions to sleep and awakening segments in individual subjects in the control group. Table 5 shows the same information for the patient group. Transitions to sleep and awakening were scored by experts (see Section 2.1). The tables also show the time at which the subjects were awake after being placed in the MRI scanner. This part of the recording was used to validate the BOLD preprocessing pipeline. Furthermore, the initial wake epoch was defined as a separate condition in the GLM design matrix to fit the brain activations corresponding to this initial state.

### 3.2. Validation of Preprocessing Pipelines

To validate the EEG preprocessing pipeline, our unsupervised clustering algorithm introduced in [28] was applied directly to standardly preprocessed EEG data, and the resulting clusters were compared to those obtained from a large dataset also described in [28]. It was confirmed that no additional artefacts or unique clusters were found.

**Figure 4:**
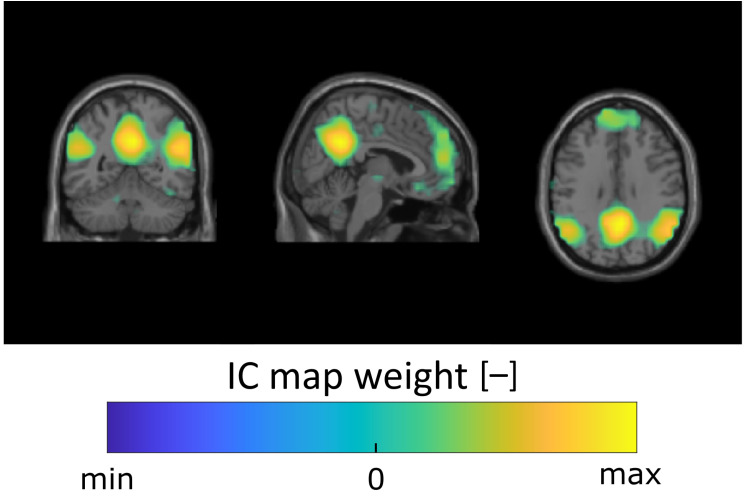
Default mode network found in the preprocessed BOLD data by spatial ICA analysis of data concatenated along the temporal domain.

To validate the BOLD preprocessing pipeline, BOLD data were merged in a time domain, and subsequently the data were subjected to a spatial ICA to determine whether resting-state networks could be found. Based on the results, a full set of resting-state networks was found in the data. Figure 4 shows the example independent component corresponding to the default mode network.

**Figure 5:**
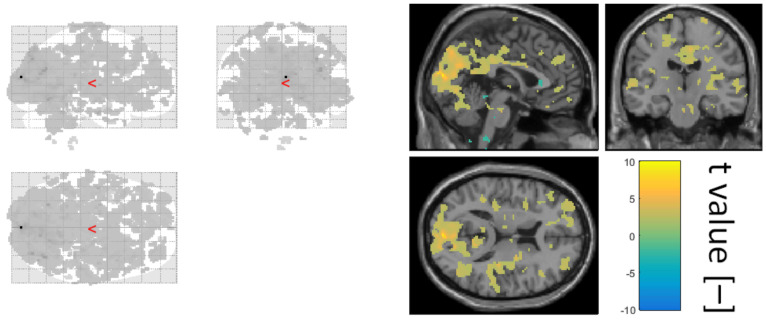
The visualised second-level statistic t values of a relative alpha power regressor. Shown using xjview at significance level 0.05.

The final step of data quality validation was to define a GLM based on a normalised alpha power time course obtained as the average over all electrodes and check whether the activation lies in the occipital part of the brain. In Figure 5, the second-level statistics over all subjects of an alpha power regressor is visualised.

### 3.3. Spectral Analysis Evaluation

**Figure 6:**
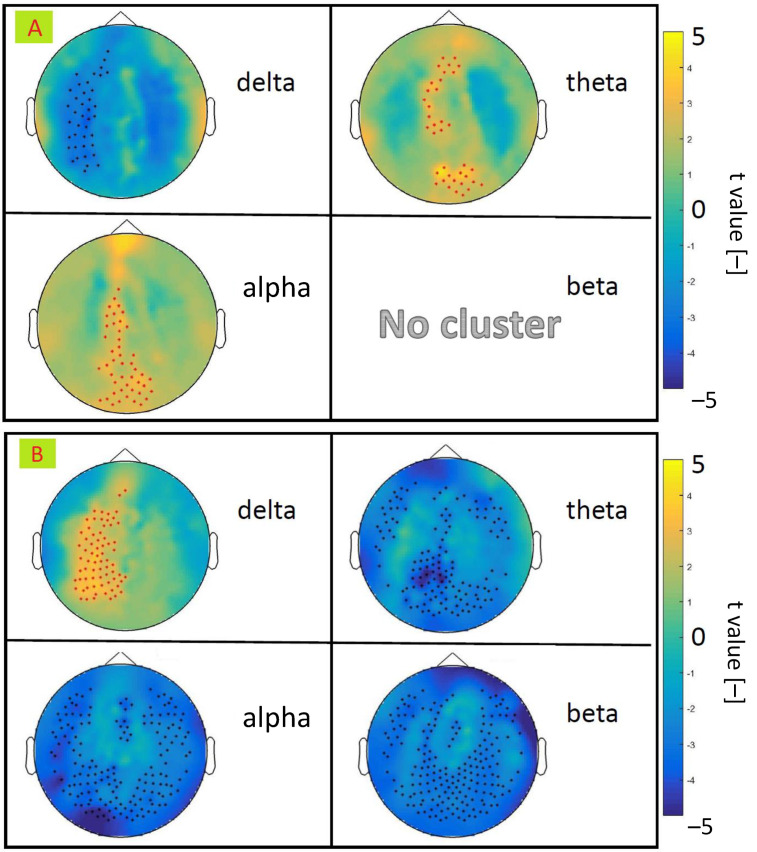
There was no significant difference between patients and controls during T epochs. Statistical differences between patient and control groups were pronounced in the delta, theta, and alpha bands (**A**). The spectral power difference between A and T epochs showed significant differences between patients and controls in all examined bands (**B**). Shown at significant level 0.05, corrected.

In the segments scored by clinicians as transitions to sleep (T) or awakening (A) described in Section 3.4, the spectral power for the four EEG bands was estimated. These values of the spectral power were normalised to the total power and subsequently entered into a nonparametric statistical analysis. Differences in spectral power between patients and the control groups were tested. Figure 6 part A depicts the electrodes showing significant differences between patients and controls in each frequency band during awakening epochs. Note that there was no significant difference of spectral power in transition to sleep epochs. Figure 6 part B depicts electrodes pronouncing significant differences between patients and controls measured by spectral power differences between awakening and transitions to sleep epochs.

Electrodes mostly contributing to the statistically significant difference between patients and controls were used to reduce the spatial dimension in the extraction of EEG covariates for GLM.

Differences were found between activations in the control group and in patients with parasomnia. Hypotheses about the difference of these groups related to epochs during sleep (T and A phase) and to fluctuations in spectral power with the BOLD signal were tested; see summarised hypotheses in Section 2.9.

### 3.4. A and T Epochs

The A and T conditions were indicated by physicians for patients and controls. Each record contained a different number of these epochs and their different durations. A summary of these parameters is described in Table 6.

**Figure 7:**
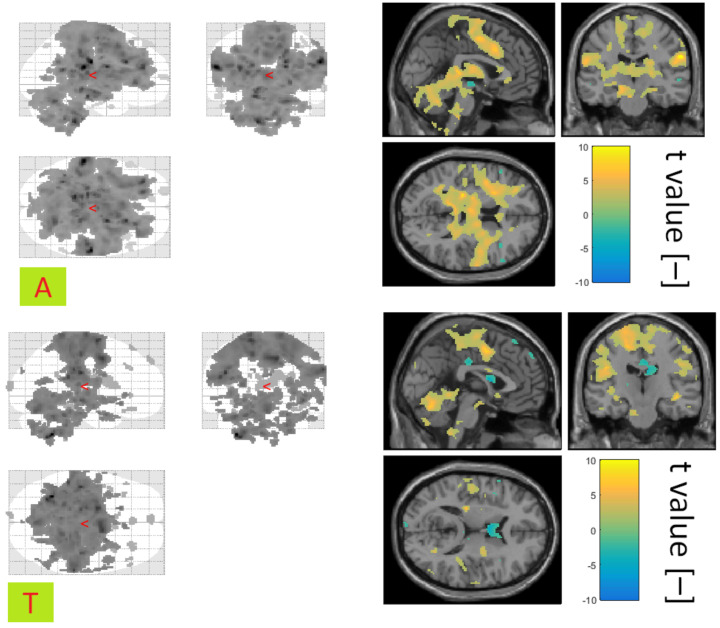
Activation associated with the A (**top**) and T (**bottom**) conditions for the control group. Glass Brain (**left**) and a selected slice with the display of the colour scale of t values (**right**). Shown using xjview at significance level 0.05.

The covariates of awakening and transition to sleep in the control group showed mostly positive correlations with the BOLD signal. Activations associated with condition A showed a symmetrical distribution in the brain and an overall broader spatial representation than for condition T (see Figure 7). The jointly activated areas for condition A and T in the control group were the thalamus and the frontal lobe.

**Figure 8:**
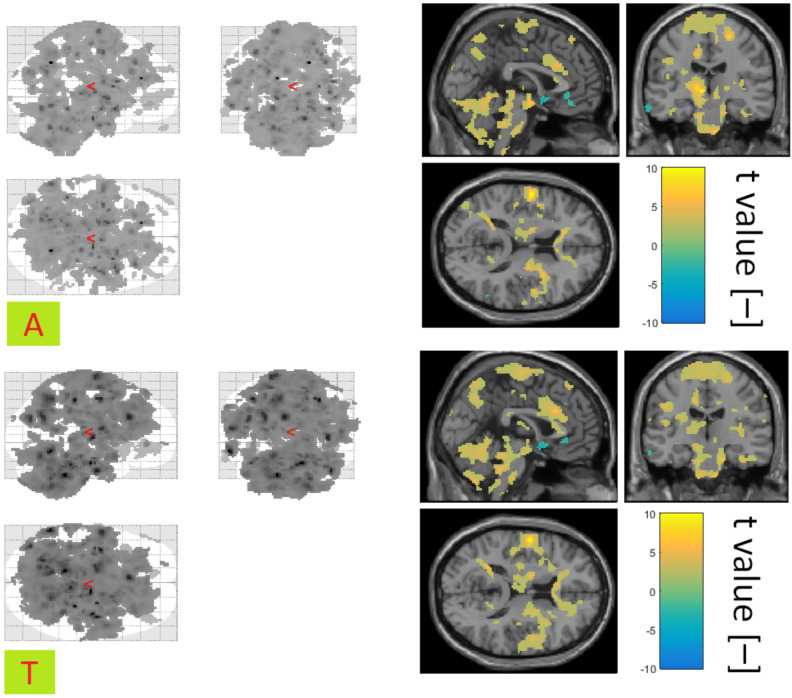
Activation associated with the A (**top**) and T (**bottom**) conditions for the patient group. Glass brain (**left**) and a selected slice displaying the colour scale of t values (**right**). Shown using xjview at significance level 0.05.

In the group of patients, fewer areas were activated than in the control group under condition A. At the same time, more activations were registered for the T condition than A in the group of patients (see Figure 8). Common activations for both conditions were observed in the patient group in the area of the Frontal and Precentral Gyrus.

Table 7 gives an overview of the most significantly activated regions in connection with the A and T condition.

### 3.5. Band Limited Power Fluctuation

Significant changes associated with slow-wave brain activity, especially the delta and partially theta bands, are typical of parasomnia disorder. With respect to the fluctuating state between awakening and transitions to sleep and previous results (see Figure 6), the results associated with the covariate representing power fluctuations in the alpha band were also included. Table 8 lists the areas of the most significant activations associated with a given frequency band for the band limited power (BLP) fluctuation conditions.

Activations associated with power fluctuations in the alpha band showed spatially similar characteristics, but with the opposite direction of correlation. The precuneus, secondary visual cortex, and dorsal posterior cingulate cortex areas were activated for both control and patient groups.

Under the conditions of spectral power fluctuations in the theta band, a lower number of activations was generally observed compared to activations associated with the alpha band. The opposite trend in correlation (precuneus, secondary visual cortex, and dorsal posterior cingulate cortex) was observed again. Co-activation for both groups of patients and controls was detected in the dorsal prefrontal cortex region.

The covariate representing power fluctuations in the delta band and in the theta band showed a small amount of significant correlation with the BOLD signal in the control group. On the other hand, more areas associated with this covariate were involved in the group of patients. The precuneus was not associated with the control group, and in the patient group there was a negative correlation.

The precuneus, secondary visual cortex, and dorsal posterior cingulate cortex are associated with parasomnia in the literature [10], so these areas have been investigated in relation to significant activations and their direction of t values (direction of correlation) (see Table 9).

## 4. Discussion

The goal of our study was to gain knowledge of NREM parasomnia disorders utilising concurrently registered EEG and fMRI signals. The purpose of this paper was to assess simultaneous EEG and fMRI data acquisition, signal integration, and statistical analysis for the characterisation of a specific sleep disorder called NREM parasomnia. The proposed methodology was adapted on the very unique dataset. The complexity of the measured dataset was amplified by the sleep disorder of the tested patient group; see discussion below. In this section, we discuss the obtained results, our data analysis pipelines, and some limitations of the experiment conducted.

**Figure A1:**
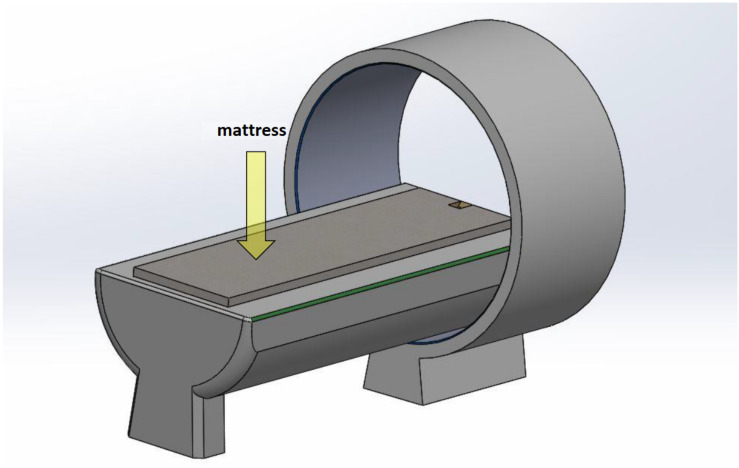

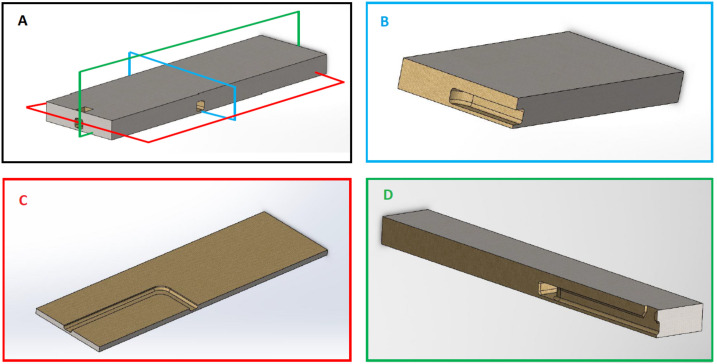
Design of the solution to the most significant discomfort, preventing the transition and maintaining deep sleep during simultaneous HD EEG and fMRI measurements. In the upper left corner, the mattress is removed out from the MR scanner table; the individual views **B**, **C**, and **D** correspond to the colours indicated in **A**.

The first observation was that, despite the control of subject comfort, most of the recordings did not contain enough NREM sleep phases. There was no technical limitation in this context, but the main methodology bottleneck was still the subject comfort. The main sources of discomfort were the increasing pressure of hdEEG net occipital electrodes (see Figure 3), and the pressure of the cable harness placed under the subject’s body. The former problem was difficult to solve due to limited space in the receiving MR head coils, and the latter requires significant changes in the MRI table to be fully eliminated. Furthermore, an ideal technical setup would entail a cable harness lying under the subject’s body parallel to the z axis of the scanner. This leads to conflicting criteria. In light of the discomfort caused by cable pressure and the orientation of the cable bundle in the main magnetic field with regard to minimising the induction of the artefact of this field, we propose a solution using a mattress with viscous foam; see Figure A1.

Although all subjects filled in the questionnaire before recording that they do not suffer from claustrophobia, three out of twenty recorded subjects confirmed a first case of claustrophobic sensations after the experimental measurement. It is therefore appropriate to include habituation to the measuring inside the MR scanner before the experimental part.

The previous simultaneous EEG–fMRI studies on sleep did not solve or discuss the subjects’ comfort in appropriate detail. We strongly recommend solving the comfort issue in future studies systematically. Further research utilising simultaneous EEG and fMRI should consider the limitations mentioned above in advance to the protocol design.

Regarding the mentioned limitations, there were an insufficient amount of deeper sleep stages for appropriate statistics. The deep sleep stage is associated with the occurrence of abnormal activity in parasomnic disorders [7,42,43]. However, repeated transitions to sleep and awakening events were present in all subjects; see Table 4 and Table 5. Segments corresponding to transitions to sleep and awakening were compared between the control group and the DOA patients for this reason. This was a unique way of tackling with otherwise underpowered GLM statistics. In this way, enough statistical power was obtained for finding activated brain areas. We have proposed two approaches to EEG and BOLD signal integration. In the first place, the EEG data were scored by experts to obtain events related to sleep. These marked events were considered as EEG correlates of sleep and informed the fMRI analysis. An advantage of this approach lies in considering only specific events corresponding to sleep as regressors of the BOLD signal. Second, the BOLD covariates were defined as an average of statistically significant electrodes between patients and controls defined (see Figure 6) for each EEG band spectral power. The EEG-based regressors were considered as a continuous time series lasting from the beginning to the end of each session.

It was found that EEG-based GLM analysis informed by preceding spectral analysis is more sensitive compared to an averaging across all electrodes. Finally, it should be mentioned that more statistical power (more subjects) is essential for future studies to obtain a more sensitive integration pipeline.

The spectral analysis was corrected for multiple comparisons, and the significance level was set at 0.05. Our results from the EEG and fMRI integration pipeline were statistically thresholded at a significance level of 0.01. The significance level set to 0.01 is strict to find areas of the brain with statistically significant activation. The results obtained at the significance level set to 0.01, therefore, indicate statistically more significant results. However, in the case of results with lower sensitivity, the significance level of 0.01 can lead to the suppression of interesting results. The significance level was set to 0.05 in the case of lower sensitivity, for this reason. These results were not corrected for multiple comparisons. Despite the low sensitivity of our statistics, we considered the obtained results unique enough for broader discussion. Furthermore, there are studies in the open literature [44,45,46] that also report results obtained without correction for multiple comparisons.

Using our previously proposed method [28], we checked whether artefact residues remain in the EEG data. We found that there is significant residue from the cardioballistic artefact, and there are also remnants of electrooculographic and electromyographic artefacts. However, the found residues did not deviate from the standardly preprocessed recordings reported in [28]. In the future, it would be appropriate to consider the use of a methodology [28] to exclude detected clusters containing residues from further analysis of the EEG signal. This study points to the need to monitor the quality of data that enters statistical analyses. This step is not common practice in clinical and experimental EEG–fMRI reports.

Resting state networks were found in the BOLD signal and were used to evaluate the effectiveness of selected steps of fMRI data preprocessing in this study. Resting state networks are usually a reflection of resting activity. The found network (see Figure 4) corresponds to the standard symmetric activations of rest DMN [47]. DMN is not primarily used to evaluate data quality. However, we consider the DMN as an effective evaluation tool since it is a well established fMRI concept in the literature.

During the awakening phase for both groups, the frontal lobe area associated with motor tasks is significantly activated. Furthermore, the thalamus, cingular gyrus, and hippocampus are significantly active, too. The most significant differences between patients with NREM parasomnia and healthy controls have been registered in anterior prefrontal cortex. Anterior prefrontal cortex works as a switch, which allows us to keep a previously running task in the waiting phase so that it can be subsequently invoked [48]. Anterior prefrontal cortex is negatively correlated in the patient group, so neuronal synchronisation can be expected.

During the transition to sleep phase in healthy individuals ventral anterior cingulate cortex, secondary visual cortex, and the motoric part of the frontal lobe was significantly activated. Ventral anterior cingulate cortex is associated with emotions [49], and secondary visual cortex is associated with visual images processing [50], which could reflect the remaining conscious processing of experiences and thoughts. In patients with NREM parasomnia, the insula and precuneus areas were significantly activated. The middle temporal gyrus, inferior frontal gyrus, and dorsal anterior cingulate cortex were significantly activated too. The inferior frontal gyrus is associated with language function [51], which could although reflect the remaining conscious processing of experiences and thoughts, as in the control group. In the study [52], when measuring fMRI in sleep-deprived subjects, compensatory reactions were also connected with middle temporal gyrus. Our results show that the compensation mechanism was more pronounced in the group of patients with NREM parasomnia.

Premotor cortex was similarly activated for patients and controls at the significance level of 0.01. This area is part of the premotor cortex and is responsible for conscious movements [53]. Significant differences between patients and controls were also observed in dorsolateral prefrontal cortex, which is part of the motor cortex. The frontal cortex was already associated with different perfusions between healthy and parasomnic individuals in the literature [54]. In the literature [42,43], awakening is indicated as one of the moments related to the knowledge of NREM parasomnia. Our results show that the mechanism of transition in the opposite direction, from waking to sleep, could be also disrupted by the different behaviour of the frontal motor part of the cortex in patients with NREM parasomnia.

The precuneus, secondary visual cortex, and dorsal posterior cingulate cortex were the most pronounced areas related with BLP covariates. These areas have been shown to be significant in a recent study of parasomnia from 2016 [10]. In Table 9, it is evident that the precuneus is positively correlated with alpha and theta covariates in the control groups, while in patients with NREM parasomnia, the correlation is negative. This could indicate the synchronisation of neurons in this region and thus increased theta and alpha spectral activity. Delta BLP covariates do not manifest in the precuneus at all in the control group, and for dorsal posterior cingulate cortex it is also the only different BLP band, because it correlates positively in the controls and negatively in the patient group. Secondary visual cortex has the opposite direction of correlation for all bands: positive for the control group and negative for the patients.

Finally, the activations for transition to the sleep and awakening phases in patients and controls were investigated in these areas: the precuneus, secondary visual cortex, and dorsal posterior cingulate cortex. The only significant, positively correlated activations were recorded in the group of patients with NREM parasomnia for the precuneus in both phases. The precuneus is involved in defining the spatial location of objects in relation to the body, receiving visual inputs from the visual cortex, stimuli related to the relationship between the hand and objects beyond and within distance, and determining the location of sound in space [55]. Therefore, the precuneus is strongly involved in the analysis and integration of visual, audio, and somesthetic information. At the same time, the precuneus is able to monitor movements in various parts of the body [56]. Thus, it could be assumed that complex motor and cognitive processes, which may appear significantly during a sleepwalking attack, may be associated with the precuneus. This fact explains the orientation in space, sound, and senso-motor perceptions patients with NREM parasomnia.

The promising results of this study are a prerequisites for further investigation. In future work, we want to verify our results on the larger dataset and extend the study condition. We will focus on improving the technical aspects of measuring, based on the results of this study.

## 5. Conclusions

The methodology for simultaneous (EEG and fMRI) sleep recording was proposed. It was not possible to induce physiological sleep in the tested subjects (neither complete sleep cycle took place) mostly due to the discomfort of the subjects in the MRI scanner. For this reason, a new data analysis pipeline was proposed based on analysis of the fluctuations of the transitions to awakening and sleep phases. The proposed methodology was adapted to compare the group of DOA patients with the control group, and we reported technical and physiological limitations. A unique dataset of simultaneous EEG and fMRI data of adults suffering from parasomnia was obtained. Spectral analysis revealed that patients with NREM parasomnia differed from the control group in the awakening phase, when, in addition to the physiological increase in alpha band power, significant delta and theta band activity was still propagated. In the GLM analysis, differences in the deepening of sleep could be observed in our dataset in terms of spatially more significant activations in a group of patients. An intersection method was proposed for a between-group comparison of EEG-informed GLMs. The secondary visual cortex and dorsal posterior cingulate cortex were found to be associated with a negative alpha BLP correlation and with the precuneus with a negative delta BLP correlation, specifically in the patients and not in the controls. Our results indicate significant activations of the motor cortex and the occipital region during fluctuating sleep in individuals with NREM parasomnia. At the same time, delta activity was promoted in these individuals, which was not represented in the control group.

## Figures and Tables

**Table 1:** Exclusion criteria of volunteers before starting the measurements.

Exclusion Criteria before Start the Experiment
pregnant or breastfeeding women
persons taking medications that affect sleep
persons with clinically significant sleep comorbidities
persons with clinically significant neurological or psychiatric comorbidities
persons who cannot be examined by a MRI device due to somatic or mental illness

**Table 2:** GES 400 system parameters.

Species of Equipment	System Version
Firmware	2.0.14
Amp Server	3.6.0
FPGA	7.40
HydroCel GSN	220 MR LTM

**Table 3:** fMRI sequence parameter settings.

Parameter	Setting
Field of view (FoV)	220 mm
Resampling phase (FoV)	100%
Slice thickness	3 mm
Time echo (TE)	30 ms
Repetition time (TR)	1000 ms
Voxel size	3 mm × 3 mm × 3 mm
SNR	1
Multi-band acceleration factor	4
Number of slices	64
Maximal numb. of sken	4960
Flip angle	52 ${}^{\circ }$
Band width	1988 Hz/Px
Echo gap	0.58 ms

**Table 4:** The duration for which the subject was awake after being placed in the MRI scanner in seconds, the total duration, and number of transitions to sleep and awakening events for every subject in the control group.

ID Control	Start Wake	Transitions to Sleep		Awakening	
	Time [s]	Count [-]	Time [s]	Count [-]	Time [s]
1	20	96	331	64	187
2	17	9	38	13	30
3	27	10	53	85	1043
4	25	20	39	49	208
5	30	13	44	10	31
6	27	22	89	31	85
7	30	15	44	41	716
8	25	9	16	9	22

**Table 5:** The duration for which the subject was awake after being placed in the MRI scanner in seconds, the total duration, and number of transitions to sleep and awakening events for every subject in the patient group.

ID Patient	Start Wake	Transitions to Sleep		Awakening	
	Time [s]	Count [-]	Time [s]	Count [-]	Time [s]
1	20	11	31	14	26
2	20	17	84	28	115
3	16	7	20	30	143
4	18	10	32	7	76
5	20	9	37	23	70
6	21	14	70	19	76
7	29	3	5	47	693
8	34	4	26	35	348
9	17	7	31	6	16

**Table 6:** Information about the duration of stages A and T for patients.

Parameter of Occurrence	Patient		Control	
	T	A	T	A
Average duration per occurrence [s]	6.2	3.9	5.7	3.5
Median of duration per occurrence [s]	4.1	4.1	3.1	3.4
Variance of duration per occurrence [$s^{2}$]	2.1	20.2	1.3	29.4

**Table 7:** Information about the main activation of the A and T conditions.

Control	
**A**	**T**
Cingular gyrus	Cingular gyrus
Hipocampus	Dorsolateral prefrontal cortex
Primary motor cortex	Secondary visual cortex
Supramarginal gyrus	Ventral anterior cingulate cortex
**Patient**	
**A**	**T**
Anterior prefrontal cortex	Insula
Supramarginal gyrus	Precuneus
Inferior frontal gyrus	Middle temporal gyrus
	Dorsal anterior cingulate cortex
	Inferior frontal gyrus

**Table 8:** Information about the main activation of the spectral fluctuation conditions.

Control		
alpha	theta	delta
Midbrain	Dorsolateral prefrontal cortex	Thalamus
Secondary visual cortex	Anterior prefrontal cortex	Secondary visual cortex
	Supramarginal gyrus	Retrosplenial cortex
	Auditory cortex	Dorsal anterior cingulate cortex
**Patient**		
alpha	theta	delta
Cerebellum	Dorsolateral prefrontal cortex	Parahipocampus
Precuneus	Insular cortex	Frontal eye fields
Secondary visual cortex	Secondary visual cortex	Dorsolateral prefrontal cortex
Dorsal posterior cingulate cortex	Supramarginal gyrus	Secondary visual cortex
		Associative visual cortex
		Supramarginal gyrus

**Table 9:** Information about the correlation direction of significant activation of spectral fluctuation conditions.

Area	Band	Control	Patient
	alpha	+	-
Secondary visual cortex	theta	+	-
	delta	+	-
	alpha	+	-
Precuneus	theta	+	-
	delta	no activation	-
	alpha	+	+
Dorsal posterior cingulate cortex	theta	+	+
	delta	+	-

## Abbreviations

The following abbreviations are used in this manuscript:

AAL	automated anatomical atlas
AAS	average artefact subtraction
ADJUST	Artifact Detector based on the Joint Use of Spatial and Temporal Features
BOLD	blood-oxygen-level-dependent
EEG	electroencephalography
fMRI	functional magnetic resonance imaging
GA	gradient artefact
GLM	general linear model
ICA	independent component analysis
MARA	multiple artefact rejection algorithm
NREM	non-rapid eye movement
OBS	optimal basis set
PCA	principal component analysis
PA	pulsion artefact
REM	rapid eye movement
SASICA	semi-automatic selection of independent components for artefact correction
t-SNE	t-distributed stochastic neighbour embedding
